# Construction and Validation of a Risk Prediction Model for Acute Kidney Injury in Patients Suffering from Septic Shock

**DOI:** 10.1155/2022/9367873

**Published:** 2022-01-06

**Authors:** Suru Yue, Shasha Li, Xueying Huang, Jie Liu, Xuefei Hou, Yufeng Wang, Jiayuan Wu

**Affiliations:** ^1^Clinical Research Service Center, The Affiliated Hospital of Guangdong Medical University, Zhanjiang, 524001 Guangdong Province, China; ^2^Collaborative Innovation Engineering Technology Research Center of Clinical Medical Big Data Cloud Service in Medical Consortium of West Guangdong Province, The Affiliated Hospital of Guangdong Medical University, Zhanjiang, 524001 Guangdong Province, China

## Abstract

**Background:**

Acute kidney injury (AKI) is an important complication in critically ill patients, especially in sepsis and septic shock patients. Early prediction of AKI in septic shock can provide clinicians with sufficient information for timely intervention so that improve the patients' survival rate and quality of life. The aim of this study was to establish a nomogram that predicts the risk of AKI in patients with septic shock in the intensive care unit (ICU).

**Methods:**

The data were collected from the Medical Information Mart for Intensive Care III (MIMIC-III) database between 2001 and 2012. The primary outcome was AKI in the 48 h following ICU admission. Univariate and multivariate logistic regression analyses were used to screen the independent risk factors of AKI. The performance of the nomogram was evaluated according to the calibration curve, receiver operating characteristic (ROC) curve, decision curve analysis, and clinical impact curve.

**Results:**

A total of 2415 patients with septic shock were included in this study. In the training and validation cohort, 1091 (64.48%) of 1690 patients and 475 (65.52%) of 725 patients developed AKI, respectively. The predictive factors for nomogram construction were gender, ethnicity, congestive heart failure, diabetes, obesity, Simplified Acute Physiology Score II (SAPS II), angiotensin-converting enzyme inhibitors (ACEI) or angiotensin receptor blockers (ARBs), bilirubin, creatinine, blood urea nitrogen (BUN), and mechanical ventilation. The model had a good discrimination with the area under the ROC curve of 0.756 and 0.760 in the training and validation cohorts, respectively. The calibration curve for probability of AKI in septic shock showed optimal agreement between prediction by nomogram and actual observation. Decision curve and clinical impact curve analysis indicated that the nomogram conferred high clinical net benefit.

**Conclusion:**

The proposed nomogram can quickly and effectively predict the risk of AKI at an early stage in patients with septic shock in ICU, which can provide information for timely and efficient intervention in patients with septic shock in the ICU setting.

## 1. Introduction

Septic shock is a life-threatening severe disease caused by circulatory and cellular metabolic abnormalities; it affects 10%–30% of patients in the intensive care unit (ICU) [1, 2]. The mortality rate of septic shock is estimated to be 45%–63% [3]. Septic shock is the most common cause of acute kidney injury (AKI) in critically ill patients. The decreased renal blood flow, secondary tubular epithelial cell death, or acute tubular necrosis is the core mechanisms underlying AKI in septic shock patients [4, 5]. The prevalence of AKI in patients suffering from septic shock was up to 60.47%, and the mortality rate is as high as 62.1% [5, 6]. In addition, the development of AKI during septic shock increases the patients' mortality and prolongs the hospital stay [7, 8]. However, AKI can be prevented in the early stage of septic shock because of the compensatory and reverse recovery function of the kidneys [9]. Thus, a simple and convenient method can be used to quickly assess the risk of AKI in septic shock.

For the past decades, many researchers have focused on the risk factors of AKI in patients suffering from septic shock. Among them, blood or serum biomarkers, such as the delta neutrophil index (DNI), proenkephalin (PENK), urinary interleukin-18, urinary KIM-1, and neutrophil gelatinase-associated lipocalin (NGAL), have attracted attention [10–14]. However, these indicators are difficult to apply to clinical practice due to the high cost and technical requirement. Moreover, several risk factors of AKI development in critically ill patients, including older age, obesity, mechanical ventilation, low white blood cell (WBC), and platelet counts, have been explored [15]. However, these indices are not stable when they solely acted as a single index due to the effects of confounding factors. Thus, it is more appropriated to incorporate them into a comprehensive model. A nomogram is a user-friendly tool with graphical representation that can be used to calculate the probability of a specific event for each individual. Compared with single indexes, nomograms can more accurately estimate the risk of AKI for individual patients by incorporating multiple risk factors. Recently, several nomograms have been developed for the prediction of AKI in many diseases. Deng et al. incorporated the information of 2917 patients with sepsis, including blood urea nitrogen (BUN), infusion volume, serum lactate, weight, serum chloride, body temperature, and age, to formulate a nomogram to predict AKI during the first 24 h of ICU stay, and the model showed excellent performance with a *C* index of 0.80 [16]. A nomogram using the routine information in ICU was well-calibrated and clinically useful for the prediction of AKI in patients with diabetic ketoacidosis [17]. However, nomograms for predicting AKI in patients suffering from septic shock are yet to be reported. As the high mortality rate of AKI in septic shock is partly due to a delay in diagnosis, identifying the septic shock patients at high risk of AKI can help clinicians take timely and effective intervention measures, reduce the mortality, and improve the quality of life. Therefore, this study is aimed at developing and validating a nomogram for predicting AKI in patients suffering from septic shock in the ICU.

## 2. Methods

### 2.1. Source of Data

The data of this study was extracted from the Medical Information Mort for Intensive Care III (MIMIC-III) database [18]. MIMIC-III is a large, free accessible intensive care database that contains the detailed information of more than 40,000 patients admitted to the critical care units in the Beth Israel Deaconess Medical Center (BIDMC) from 2001 to 2012, including demographic characteristics, monitoring vital signs, laboratory and microbiological examination, imaging examination, observation and recording of intake and output, drug treatment, length of stay, survival data, and discharge or death records. To apply for access to the database, we passed the protection of human research participant examination and obtained the certificate (No. 9983480). Structured Query Language (SQL) was used to extract all patients' information from the MIMIC-III database.

### 2.2. Participants

Inclusive criteria were as follows: (1) first admission to ICU and (2) diagnosis of septic shock upon admission. Exclusion criteria were as follows: (1) the length of stay in ICU was less than 48 h, (2) ≤18 and ≥89 years old, (3) underwent renal replacement therapy or continuous renal replacement therapy, (4) died within 48 h after ICU admission, and (4) diagnosed with end-stage renal disease or chronic kidney disease upon admission. For patients older than 89 years, the actual age could not be obtained due to the date of birth being 300 years before the first admission [19]. Thus, our study excluded age over 89 years old.

### 2.3. Diagnosis of Septic Shock and AKI

According to the Third International Consensus Definitions (ICDs) for Sepsis and Septic Shock (Septic-3), septic shock was defined as an infection or a suspected infection with a vasopressor requirement to maintain a mean arterial pressure of 65 mmHg or greater and serum lactate level of >2 mmol/l (18 mg/dl) in the absence of hypovolemia [20]. In the present study, we identified patients suffering from septic shock based on the ICD-9 (78552). The primary outcome of this study was the development of AKI within 48 h after ICU admission. A diagnosis of AKI was made according to the acute kidney injury network (AKIN) criteria [21], including the increase of serum creatinine ≥ 26.5 *μ*mol/l or 1.5 times higher than baseline within 48 h or urine out < 0.5 ml/(kg·h) for more than 6 h. The reasons for the use of AKIN rather than KDIGO criteria for AKI diagnosis in this study are as follows: (1) the data recorded by the MIMIC-III database were earlier than the release time of KDIGO criteria and (2) KDIGO criteria were based on the changes of kidney function within 7 days, but many confounding factors, such as antibiotics and nosocomial infection, may affect renal function [22].

### 2.4. Research Variables

Data of each patient, including demographic characteristics, comorbidity, vital signs, interventions, and laboratory examinations, were obtained from the MIMIC-III database [23, 24]. Demographic characteristics, including age, gender, and race, were collected from the original database. Vital signs, including respiratory rate (beats/min), heart rate body (beats/min), and temperature (°C) upon ICU admission, were collected from charting in CHARTENS. Laboratory parameters, including WBC count, hemoglobin, platelet count, BUN, potassium, bicarbonate, chloride, neutrophils, lymphocytes, and lactic acid, were recorded in the table of laboratory events. Interventions, including mechanical ventilation, angiotensin-converting enzyme inhibitors (ACEIs), and angiotensin receptor blockers (ARBs), were recorded in the first 24 h after admission. Acute physiology score III (APS III) and Simplified Acute Physiology Score (SAPS II) were calculated using the data of the first 48 h of the ICU stay. All comorbidities were identified according to the ICD-9 code records. Variables with >20% missing values were excluded from further analysis, and variables with ≤20% missing values were filled with the multiple imputation.

### 2.5. Statistical Analysis

All eligible patients were divided into the training and validation cohorts with the split ratio of 7 : 3. The data of the training cohort were used to perform the logistic regression analysis and construct the nomogram, whereas the data in the validation cohort were used to validate the model. Continuous variables were expressed as median with quartile. These data were compared by *t*-test or rank sum test, as appropriate. *χ*^2^ test or Fisher's exact test was used to compare the categorical variables. Potential multicollinearity between variables was judged by the variance expansion factor (VIF). A VIF of ≥5 was considered evidence of multicollinearity [25]. Univariate and multivariate logistic regression models were used to select the independent risk factors of AKI in the training cohort. According to the results of multivariate logistic regression analysis, the nomogram was developed to predict the risk of AKI in patients suffering from septic shock in the ICU. The performance of the nomogram was first quantified in the training cohort and then in the validation cohort in terms of discrimination, calibration, and clinical utility. The discrimination of the nomogram was evaluated by the area under curve (AUC) of the receiver operating characteristics (ROC) curve. The calibration curve was drawn to examine the consistence of the predicted probabilities and the observed outcomes. The clinical applicability of the nomogram was estimated by the decision curve analysis (DCA) and clinical impact curve. The statistical significance of all analyses was set at a *P* level less than 0.05. All analyses were performed using R version 4.0.5 (http://www.r-project.org/).

## 3. Results

### 3.1. Baseline Characteristics

**Figure 1:**
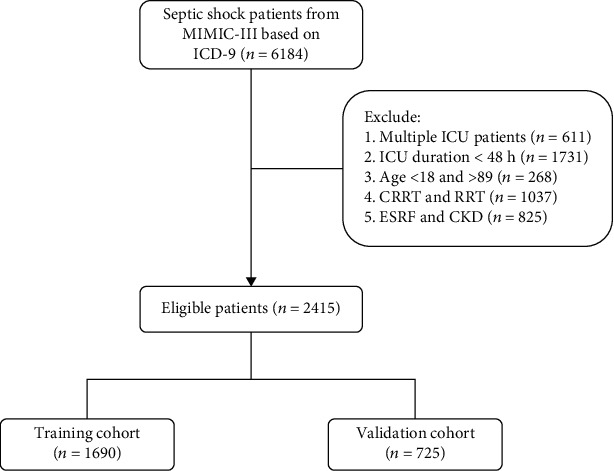
The flowchart of patient selection. MIMIC-III: Medical Information Mort for Intensive Care III; ICU: intensive care unit; CRRT: continuous renal replacement therapy.

The procedure for subject selection is shown in Figure 1. A total of 2415 patients suffering from septic shock were included in the final analysis. Among these, 1566 patients (64.84%) developed AKI in 48 h after ICU admission. Thirty-eight variables, namely, age, gender, ethnicity, obesity, congestive heart failure, hypertension, diabetes, aminoglycoside, glycopeptide antibiotics, nonsteroidal anti-inflammatory drugs (NSAIDs), stain, ACEI/ARBs, APSIII, SAPSII, heart rate, systolic pressure, diastolic pressure, respiratory rate, temperature, SpO2, anion gap, bicarbonate, bilirubin, creatinine, chloride, glucose, lactate, platelets, potassium, prothrombin time (PTT), activated partial thromboplastin time (APTT), BUN, white blood cell (WBC), neutrophils, lymphocytes, Gram-positive bacteria, Gram-negative bacteria, and mechanical ventilation, were collected from the MIMIC-III database. The clinicopathological characteristics of the eligible patients are shown in Table 1. The baseline clinicopathologic data were similar between the training and testing sets. AKI was detected in 64.48% (1091/1690) and 65.52% (475/725) of the patients in the training and validation sets, respectively.

### 3.2. Predictors of AKI and Nomogram Development

**Figure 2:**
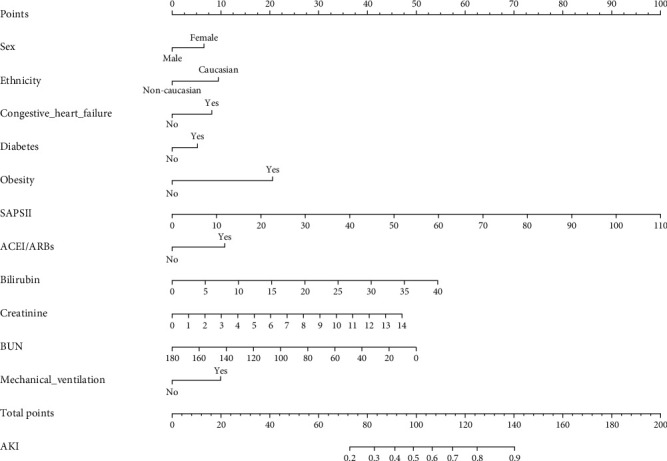
Nomogram to identify the risk of AKI in septic shock, based on logistic regression analysis. To acquire the corresponding scores for each variable, draw a vertical line upward to the “Points” axis. Sum the score for all predictors and locate the final value on the “Total Points” axis. Draw a line straight down to the “Probability of AKI” axis to determine the risk of AKI. Abbreviations: AKI: acute kidney injury; SAPS II: Simplified Acute Physiology Score II; ACEI: angiotensin-converting enzyme inhibitors; ARBs: angiotensin receptor blockers; BUN: blood urea nitrogen.

After multicollinearity examination, no multicollinearity was found between variables, because all VIFs were less than 5 (Table 2). Thus, all 38 features were included in the univariate logistic regression analysis. Based on the univariate logistic regression analyses, 11 variables, namely, gender, race, congestive heart failure, diabetes, obesity, SAPS II, ACEI/ARBs, bilirubin, creatinine, BUN, and mechanical ventilation, were significantly associated with AKI development in patients suffering from septic shock (Table 3). In multivariate analysis, gender, race, congestive heart failure, diabetes, obesity, SAPS II, ACEI/ARBs, bilirubin, creatinine, BUN, and mechanical ventilation were identified as the independent risk factors for AKI in patients suffering from septic shock (*P* < 0.05, Table 4). Therefore, a nomogram for predicting AKI in patients suffering from septic shock was constructed based on these variables (Figure 2).

### 3.3. Nomogram Validation

**Figure 3:**
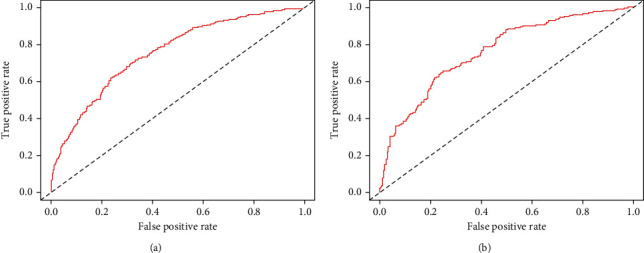
Receiver operating characteristic curve of the nomogram. Receiver operating characteristic curve for predicting AKI in septic shock patients during the intensive care admission. AUC: area under the receiver operating characteristic curve. The AUC of the nomogram for the prediction of AKI in septic shock patients was 0.756 in the training set and 0.760 in the validation set.

**Figure 4:**
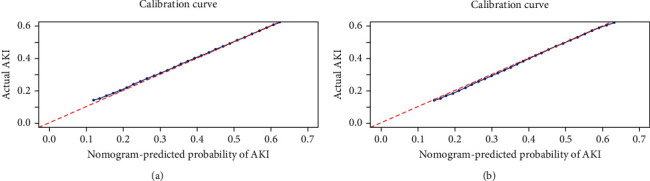
Calibration curves of the predicted nomogram in the training set (a) and validation set (b). The *x*-axis represents the predicted probability calculated by the nomogram, and the *y*-axis is the observed actual probability of AKI. The clinodiagonal represents a perfect prediction by an ideal model. The solid curve represents the initial cohort and the dotted curve is bias corrected by bootstrapping (*B* = 1000 repetitions), which demonstrates the performance of the predicted model.

**Figure 5:**
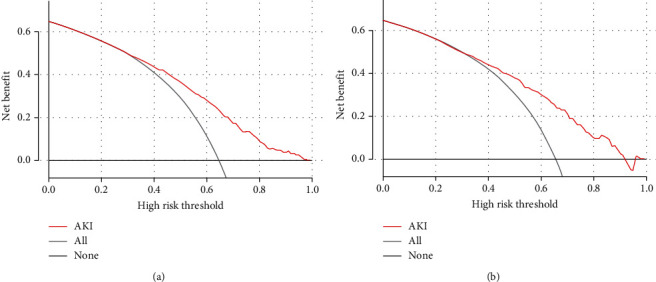
Decision curve analysis (DCA) of the nomogram in the training set (a) and the validation set (b). The horizontal line indicates no patients develop acute kidney injury (AKI), and the gray oblique line indicates patients develop AKI. The red solid line represents the AKI risk nomogram. In DCA, the nomogram shows a more net benefit than full or no treatment across a threshold probability range. DCA: decision curve analysis; AKI: acute kidney injury.

**Figure 6:**
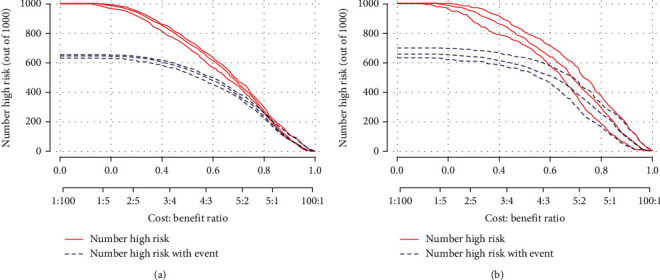
Clinical impact curve (CIC) of nomogram. The red curve (number of high-risk individuals) indicates the number of people who are classified as positive (high risk) by the model at each threshold probability; the blue curve (number of high-risk individuals with outcome) is the number of true positives at each threshold probability. CIC visually indicated that nomogram conferred high clinical net benefit and confirmed the clinical value.

First, the ROC curves are shown in Figure 3, and the discriminant results showed that the model has a good ability to distinguish AKI patients from non-AKI patients with AUCs of 0.756 and 0.760 in the training and validation cohorts, respectively. Second, the predictive probabilities of the model were in consistent agreement with the observation results in both the training and validation sets, thereby suggesting a good calibration (Figure 4). Third, the DCA (Figure 5) and clinical impact curve analysis (Figure 6) visually showed that the nomogram had superior overall net benefit within the wide and practical ranges of threshold probabilities and impacted patient outcomes, thereby indicating that the nomogram had significant predictive value. When the predicted probability thresholds were set as 30%–100% and 30%–93% in the training and validation cohorts, the net benefit ranges were 0%–50% and 0%–47%, respectively. The smaller the threshold was, the better the net benefit was.

## 4. Discussion

The incidence of AKI in septic shock patients reached as high as 64.5% in the ICU, similar to the findings of previously published studies. According to a report in Finland, a total of 488 (53.2%) AKI incidences were detected among 918 patients with severe sepsis during their stay in the ICU [26]. Another study found that AKI developed in 572 (57.7%) of the 992 patients with sepsis and septic shock patients [15]. However, the incidence rate of AKI was higher in patients suffering from septic shock than in patients suffering from other diseases. For example, the incidence of AKI was 40%–50% among patients with sepsis in the ICU [5]. Fan et al. reported that AKI was developed in 41.3% (314/760) of the patients with diabetic ketoacidosis after ICU admission [17]. Several mechanisms might contribute to the pathogenesis of AKI in sepsis, including inflammation, microcirculatory dysfunction, and mitochondrial dysfunction [5]. The kidney is the most common organ involved in sepsis, and septic patients with kidney dysfunction have a higher risk of death than those without kidney dysfunction [27, 28]. In sepsis, circulating toxins act on the vascular endothelium, reducing microcirculation blood flow and producing a large number of inflammatory mediators, such as tumor necrosis factor and transforming growth factor *β*. Inflammatory response waterfall can lead to “intrarenal shunting,” in which the renal blood flow is shunted from the medulla to the cortex, resulting in medulla hypoperfusion even with increased renal blood flow [29]. Simultaneously, the tissue damage caused by sepsis increases the inducible nitric oxide (NO) synthase and decreases endothelial NO synthase activity. The dysfunction of NO synthase decreases nitricoxide-mediated endothelium-dependent vasodilation, resulting in the imbalance of local renal microcirculation [30]. Moreover, in sepsis, the activation of bacterial phospholipids, related cytokines, and chemical factors in the circulatory system are involved in the occurrence of tubular epithelial cell damage, leading to renal tubular necrosis, which is an important cause of AKI [29]. In addition, under the stress state caused by sepsis, mitochondrial autophagy increases to remove damaged mitochondria, thus playing a role in protecting the kidney. However, with the progress of the disease, mitochondrial autophagy is inhibited, leading to the accumulation of damaged mitochondria, thereby mediating kidney damage [31].

Logistic regression analysis showed that gender, race, congestive heart failure, diabetes, obesity, SAPS II, ACEI/ARBs, bilirubin, creatinine, BUN, and mechanical ventilation were significantly associated with AKI risk in septic shock. Compared with previous studies on sepsis-related AKI, this study had some new findings. Firstly, SAPS II score was the most sensitive indicator for predicting the risk of AKI in septic shock. SAPS II score is among the most commonly used methods to quantify the death risk of AKI patients in ICU. The higher the SAPS II score was, the greater the risk of AKI was. When patients develop AKI after ICU admission, SAPS II showed the best performance in the prediction of AKI among all scoring systems, including Acute Physiology and Chronic Health Evaluation II (APACHE II), Sequential Organ Failure Assessment (SOFA), Logistic Organ Dysfunction Score, and Organ System Failure [32]. SAPS II score was superior to APACHE II and SOFA scores, because it can predict the survival outcome in patients suffering from septic shock [33]. Secondly, hyperbilirubinemia is a common complication in septic patients; the bilirubin level in septic shock patients significantly increases in 72 h after ICU admission [34]. Hyperbilirubinemia can be found in 60% of patients with AKI [35]. Elevated serum bilirubin levels are an independent risk factor for the occurrence of AKI [36]. Elevated bilirubin may produce oxidative stress on renal tubular cells, trigger apoptosis, and aggravate renal ischemia-reperfusion injury that contributes to the development of AKI [37, 38]. Thirdly, AKI is characterized by a sharp drop in the glomerular filtration rate and a rapid increase in serum creatinine, BUN, sodium, and water storage. Serum creatinine and BUN are common indicators of renal function dysfunction. Studies have shown that even slightly elevated levels of serum creatinine and BUN are significantly associated with an increasing risk of AKI after ICU admission [39]. Moreover, modest changes of serum creatinine were significantly associated with higher mortality, longer length of hospital stay, and heavier costs in patients with AKI [39].

Other risk factors were independently associated with AKI risk, including gender, complicating diseases (diabetes, congestive heart failure, and obesity), mechanical ventilation, and ACEI/ARBs therapy. Women were more prone to develop AKI due to the effect of estradiol level on patients suffering from septic shock. Ovarian levels of estradiol at >40 pg/ml were an independent risk factor of septic shock-related AKI in women [40]. In a matched case-control study, Kim et al. found that the incidence rate of postoperative AKI was significantly higher in patients with diabetes than those without diabetes [41]. Diabetes can increase the susceptibility of renal ischemia/reperfusion injury [42]. Congestive heart failure causes the complex interaction between the heart and kidney and results in the damage of renal function. Decrease in renal perfusion is the main factor responsible for development of AKI in patients with congestive heart failure [43]. Gameiro et al. proposed that the risk of AKI development was 2.31 times higher in obese patients with sepsis than in nonobese patients [44]. A complex interplay of different mechanisms may increase the susceptibility to AKI in critically ill obese patients, such as glomerulopathy, low inflammatory status, endothelial dysfunction, enhanced oxidative stress, activation of the renin-angiotensin-aldosterone system, and increased sympathetic nervous system activity [45]. Mechanical ventilation is connected with a threefold increase in the risk of developing AKI among critically ill patients [46]. Ventilator-induced lung injury might contribute to the development of AKI during the use of mechanical ventilation [47]. Moreover, the use of ACEI/ARBs can cause efferent arteriole dilatation, decrease renal blood flow, and reduce glomerular pressure, which may increase the risk of AKI in patients suffering from septic shock [48].

In this study, we developed a simple and rapid nomogram model for predicting AKI risk in patients suffering from septic shock. The variables identified by our nomogram can be easily obtained in clinic and can reflect the disease activity of patients, thereby providing clinically relevant information in the management of patients suffering from septic shock. The proposed nomogram showed a good performance in the discrimination, calibration, and clinical application and provided valuable information for the decision-making of the appropriated therapy options for individual patients. More importantly, it filled the gap between the high incidence of AKI in septic shock and the lack of reliable predictive model. We cited an example to show how to use the nomogram model. For example, we assumed that a Caucasian man with septic shock and diabetes had received mechanical ventilation with a SAPS II score of 45, a BUN level of 80 mg/dl, a bilirubin level of 15 mg/dl, and a creatinine level of 5 mg/dl. According to Figure 2, the score corresponding to each individual parameter was obtained from the first row (the “Point” axis). For this patient, the score of gender was 0 based on the male sex, and the score of diabetes was 6 based on the complication of diabetes. Finally, the overall score was calculated as the sum of points for all parameters (0 (sex) + 10 (Caucasian) + 0 (congestive heart failure) + 6 (diabetes) + 0 (obesity) + 42 (SAPS II) + 0 (ACEI/ARBs) + 21 (bilirubin) + 18 (creatinine) + 29 (BUN) + 11 (mechanical ventilation = 137). This score corresponded to a risk of developing AKI at an approximately 87% level.

This study had several limitations. First, selection bias might be inevitable, because this study was a retrospective analysis of secondary data. Second, missing data were handled with multiple imputations, which might decrease the accuracy of the model. Third, the model was constructed based on a US population. Thus, its generalizability to the global population was still unclear. Fourth, the nomogram was developed and validated by the same database. Thus, it was more reliable to validate it prospectively or at least in another database. Fifth, because the nomogram was built based on 11 indicators, the sensitivity of the model performance may decline if the data of one or two indicators for a patient were missing. Finally, we only considered traditional parameters and did not consider some valuable biomarkers that might contribute to AKI development.

## 5. Conclusion

By incorporating 11 independent risk factors of AKI in septic shock, a simplified score model was constructed for AKI risk estimation in patients suffering from septic shock in the ICU. The proposed nomogram showed good performance in terms of discrimination, calibration, and clinical application. By early assessment of the risk of AKI development, clinicians can implement more measures that are beneficial for patients suffering from septic shock. Further studies are needed to externally validate our model using a large-sample prospective cohort study.

## Figures and Tables

**Table 1:** Baseline characteristics of the patients with septic shock.

Characteristics	Total (n =2415)	Train cohort (n =1690)	Validation cohort (n =725)	*P* value
Demographic variables				
Age (years)	65 (55-76)	65 (55-75)	65 (55-77)	0.120
Gender, *n* (%)				0.850
Male	1263 (52.30)	886 (52.43)	377 (52.00)	
Female	1152 (47.70)	804 (47.57)	348 (48.00)	
Ethnicity, *n* (%)				0.550
Caucasian	1796 (74.37)	1251 (74.02)	545 (75.17)	
Non-Caucasian	619 (25.63)	439 (25.98)	180 (24.83)	
Obesity, *n* (%)				0.020
No	2276 (94.24)	1580 (93.49)	696 (96.00)	
Yes	619 (5.76)	110 (6.51)	29 (4.00)	
Comorbidities				
Congestive heart failure, *n* (%)				0.350
No	1556 (64.43)	1099 (65.03)	457 (63.03)	
Yes	859 (35.57)	591 (34.97)	268 (36.97)	
Hypertension, *n* (%)				0.590
No	1379 (57.10)	971 (57.46)	408 (56.28)	
Yes	1036 (42.90)	719 (42.54)	317 (43.72)	
Diabetes, *n* (%)				0.660
No	1677 (69.44)	1169 (69.17)	508 (70.07)	
Yes	738 (30.56)	521 (30.83)	217 (29.93)	
Medications				
Aminoglycoside, *n* (%)				0.750
No	2266 (93.83)	1584 (93.73)	682 (94.07)	
Yes	149 (6.17)	106 (6.27)	43 (5.93)	
Glycopeptide antibiotics, *n* (%)				0.230
No	690 (28.57)	495 (29.29)	195 (26.90)	
Yes	1725 (71.43)	1195 (70.71)	530 (73.10)	
NSAIDs, *n* (%)				0.950
No	714 (29.57)	499 (29.53)	215 (29.66)	
Yes	1701 (70.43)	1191 (70.47)	510 (70.34)	
Stain, *n* (%)				0.940
No	1963 (81.28)	1373(81.24)	590 (81.38)	
Yes	452 (18.72)	317 (18.76)	135 (18.62)	
ACEI/ARBs, *n* (%)				0.820
No	961 (39.79)	675 (39.94)	286 (39.45)	
Yes	1454 (60.21)	1015 (60.06)	439 (60.55)	
Mechanical ventilation, *n* (%)				0.140
No	1138 (47.12)	787 (46.57)	351 (48.41)	
Yes	1277 (52.88)	903 (53.43)	374 (51.59)	
Scoring systems				
APS III	53 (41-68)	53 (41-67)	53 (41-68)	0.830
SAPS II	41 (32-50)	40 (31-50)	41 (32.50-51)	0.830
Vital signs				
Heart rate (beats/minute)	91 (80-104)	92 (80-104)	91 (80-103)	0.200
Systolic pressure (mmHg)	107 (100-114)	107 (100-115)	107 (100-114)	0.490
Diastolic pressure (mmHg)	57 (52-63)	57 (51-63)	57(52-63)	0.710
Respiratory rate (beats/minute)	20 (17-24)	20 (17-24)	20 (17-24)	0.630
Temperature (°C)	36.8 (36.4-37.3)	36.8 (36.4-37.3)	36.8 (36.4-37.3)	0.370
SpO2 (%)	97.4(95.9-98.7)	97.4 (95.9-98.6)	97.5 (95.9-98.8)	0.140
Laboratory test				
Anion gap (mmol/l)	14.0 (12.5-16.5)	14.0 (12.0-16.5)	14.0 (12.5-16.3)	0.950
Bicarbonate (mEq/l)	22.0 (19-25.5)	22.0 (19.0-25.5)	22.0 (18.5-25.5)	0.150
Bilirubin (mg/dl)	0.6 (0.4-1.3)	0.6 (0.4-1.3)	0.6 (0.4-1.3)	0.180
Creatinine (mg/dl)	1.1 (0.8-1.6)	1.1 (0.8-1.6)	1.1 (0.8-1.6)	0.740
Chloride (mEq/l)	105.5 (101.5-109.5)	105.5 (101.5-109.5)	106.0 (101.5-110.0)	0.150
Glucose (mg/dl)	134.5 (109.5-171)	134.5 (110.0-170.13)	135.5 (108.5-172.8)	0.790
Lactate (mmol/l)	30.9 (27.9-34.4)	1.9 (1.4-2.9)	2.0 (1.4-3.1)	0.290
Platelets (K/UL)	217.5 (137-305)	215.0 (135.5-305.0)	224.50 (143.5-306.8)	0.300
Potassium (mEq/l)	4.1 (3.8-4.6)	4.1 (3.8-4.6)	4.1 (3.8-4.6)	0.870
PTT (seconds)	34.6 (29.2-43.5)	34.7 (29.3-43.7)	34.3 (28.9-42.93)	0.210
APTT (seconds)	15.3 (13.6-18.35)	15.3 (13.7-18.5)	15.1 (13.5-18.2)	0.150
BUN (mg/dl)	24 (15.5-38.5)	24.0 (15.5-39.0)	24.5 (16.0-37.5)	0.460
WBC (K/UL)	12.7 (8.1-17.9)	12.7 (8.0-17.9)	12.70 (8.5-18.0)	0.310
Neutrophils (%)	78.9 (65.7-87)	78.7 (65.0-87.0)	79.5 (67.0-87.0)	0.460
Lymphocytes (%)	9 (5-15.4)	9.0 (5.0-15.4)	9.0 (5.0-15.5)	0.960
Culture				
Gram-positive bacteria, *n* (%)				0.300
No	1914 (79.25)	1344 (79.53)	570 (78.62)	
Yes	501 (20.75)	346 (20.47)	155 (21.38)	
Gram-negative bacteria, *n* (%)				0.490
No	2122 (87.87)	1489 (88.11)	633 (87.31)	
Yes	293 (12.13)	201 (11.89)	92 (12.69)	
AKI				0.650
No	849 (35.16)	599 (35.44)	250 (34.48)	
Yes	1566 (64.84)	1091 (64.56)	475 (65.52)	

NSAIDs: nonsteroidal anti-inflammatory drugs; ACEI: angiotensin-converting enzyme inhibitors; ARBs: angiotensin receptor blockers; APS III: acute physiology score III; SAPS II: simplified acute physiology score II; PTT: prothrombin time; APTT: activated partial thromboplastin time; BUN: blood urea nitrogen; WBC: white blood cell; AKI: acute kidney injury.

**Table 2:** Results of the multicollinearity diagnosis.

Variables	Variance expansion factor
Gender	1.13
Age	1.99
Ethnicity	1.10
Congestive heart failure	1.29
Hypertension	1.21
Diabetes	1.26
Obesity	1.13
APSIII	1.04
SAPS II	3.20
Aminoglycoside	1.08
Glycopeptide antibiotics	1.20
NSAIDs	1.17
Stain	1.17
ACEI/ARBs	1.27
Heart rate	1.55
Systolic pressure	1.68
Diastolic pressure	1.79
Respiratory rate	1.33
Temperature	1.31
SpO2	1.22
Anion gap	3.18
Bicarbonate	3.05
Bilirubin	1.29
Creatinine	2.37
Chloride	1.95
Glucose	1.29
Lactate	1.63
Platelets	1.54
Potassium	1.23
PTT	1.15
APTT	1.17
BUN	2.23
WBC	1.19
Neutrophils	1.03
Lymphocytes	1.10
Gram-positive bacteria	1.08
Gram-negative bacteria	1.12
Mechanical ventilation	1.60

NSAIDs: nonsteroidal anti-inflammatory drugs; ACEI: angiotensin-converting enzyme inhibitors; ARBs: angiotensin receptor blockers; APS III: acute physiology score III; SAPS II: simplified acute physiology score II; SBP: systolic blood pressure; DBP: diastolic blood pressure; PTT: prothrombin time; APTT: activated partial thromboplastin time; BUN: blood urea nitrogen; WBC: white blood cell; AKI: acute kidney injury.

**Table 3:** Univariate logistic regression analysis of predictive variables of AKI in the training cohort.

Variables	OR	95% CI	*P* value
Demographic variables			
Age (years)	1.01	1.17-1.89	0.340
Female, *n* (%)	1.49	0.99-1.02	<0.001
Non-Caucasian, *n* (%)	0.59	0.45-0.77	<0.001
Obesity, *n* (%)	2.61	1.02-1.76	<0.001
Comorbidities, *n* (%)			
Congestive heart failure, *n* (%)	1.34	0.95-1.57	0.030
Hypertension, *n* (%)	1.22	1.04-1.81	0.130
Diabetes, *n* (%)	1.38	1.43-4.78	0.020
Interventions			
Aminoglycoside, *n* (%)	0.99	1.00-1.01	0.960
Glycopeptide antibiotics, *n* (%)	0.81	1.03-1.06	0.130
NSAIDs, *n* (%)	0.96	0.97-1.08	0.740
Stain, *n* (%)	1.25	0.60-1.63	0.180
ACEI/ARBs, *n* (%)	1.72	0.62-1.06	<0.001
Mechanical ventilation, *n* (%)	1.87	1.41-2.47	<0.001
Scoring systems			
APSIII	1.00	0.73-1.25	0.560
SAPSII	1.05	0.91-1.71	<0.001
Vital signs	1.02	1.34-2.21	0.410
Heart rate (beats/minute)	1.01	1.00-1.02	0.230
Systolic pressure (mmHg)	1.00	0.98-1.01	0.700
Diastolic pressure (mmHg)	0.99	0.98-1.01	0.420
Respiratory rate (beats/minute)	1.00	0.97-1.03	1.000
Temperature (°C)	0.85	0.71-1.02	0.090
SpO2 (%)	0.96	0.91-1.02	0.230
Laboratory test			
Anion gap (mmol/l)	1.00	0.94-1.06	0.900
Bicarbonate (mEq/l)	1.03	0.99-1.07	0.130
Bilirubin (mg/dl)	1.07	1.03-1.13	<0.001
Creatinine (mg/dl)	1.25	1.05-1.48	0.010
Chloride (mEq/l)	0.99	0.96-1.01	0.190
Glucose (mg/dl)	1.00	0.99-1.00	0.130
Lactate (mmol/l)	1.07	0.96-1.19	0.230
Platelets (K/Ul)	1.00	0.99-1.01	0.730
Potassium (mEq/l)	1.04	0.85-1.27	0.700
PTT (seconds)	1.00	0.99-1.01	0.230
APTT (seconds)	0.99	0.98-1.00	0.050
BUN (mg/dl)	0.99	0.98-1.00	<0.001
WBC (K/Ul)	0.99	0.98-1.01	0.240
Neutrophils (%)	1.00	0.99-1.01	0.930
Lymphocytes (%)	1.00	0.99-1.01	0.310
Culture			
Gram positive bacteria, *n* (%)	1.35	1.01-1.81	0.050
Gram negative bacteria, *n* (%)	1.04	0.71-1.52	0.850

CI: confidence interval; OR: odds ratio; NSAIDs: nonsteroidal anti-inflammatory drugs; ACEI: angiotensin-converting enzyme inhibitors; ARBs: angiotensin receptor blockers; APS III: acute physiology score III; SAPS II: simplified acute physiology score II; PTT: prothrombin time; APTT: activated partial thromboplastin time; BUN: blood urea nitrogen; WBC: white blood cell; AKI: acute kidney injury.

**Table 4:** Multivariate logistic regression analysis of risk factors of AKI in the training cohort.

Variables	OR	95% CI	*P* value
Gender (female vs. male)	1.41	1.13-1.77	<0.001
Ethnicity (non-Caucasian vs. Caucasian)	0.61	0.47-0.78	<0.001
Congestive heart failure (yes vs. no)	1.53	1.19-1.97	<0.001
Diabetes (yes vs. no)	1.32	1.03-1.68	0.030
Obesity (yes vs. no)	2.98	1.66-5.34	<0.001
SAPS II	1.05	1.04-1.06	<0.001
ACEI/ARBs (yes vs. no)	1.78	1.40-2.25	<0.001
Bilirubin (mg/dl)	1.08	1.03-1.12	<0.001
Creatinine (mg/dl)	1.20	1.04-1.38	0.010
BUN (mg/dl)	0.99	0.98-0.99	<0.001
Mechanical ventilation (yes vs. no)	1.69	1.33-2.15	<0.001
Constant	0.12		<0.001

OR: odds ratio; CI: confidence interval; ACEI: angiotensin-converting enzyme inhibitors; ARB: angiotensin receptor blockers; SAPS II: simplified acute physiology score II; BUN: blood urea nitrogen; AKI: acute kidney injury.

## Data Availability

The data support the findings of this study and are available from the corresponding author upon reasonable request.
